# Supplementary material for: RNA binding by Periphilin plays an essential role in initiating silencing by the HUSH complex
Source: Nucleic Acids Res. 2024 Dec 11;53(2):gkae1165. doi: 10.1093/nar/gkae1165 (PMC11754731; doi:10.1093/nar/gkae1165)
Supplement: gkae1165_Supplemental_File [file gkae1165_supplemental_file.pdf]

Supplementary figures

Figure S1:

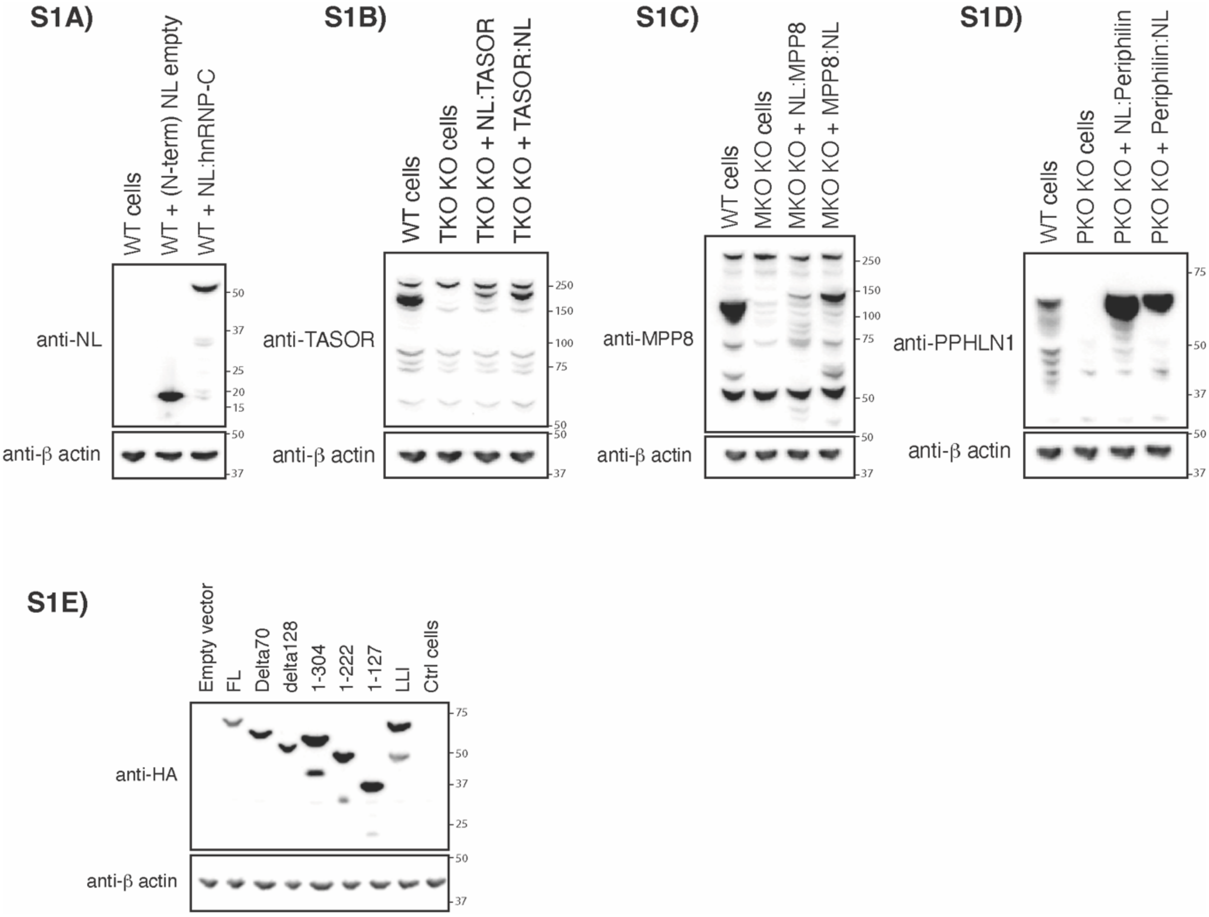

(A-D) Western blot analysis of the samples used in Fig. 1B. **(A)** Control cells, empty n-terminal NL vector and NL:hnRNP-C in wild-type cells were stained with the NanoLuc-specific mAb. **(B)** WT and TASOR KO (TKO) control cells, together with TKO cells complemented with NL:TASOR and TASOR:NL were detected with the TASOR-specific antibody. **(C)** WT and MPP8 KO (MKO) control cells, together with MKO cells complemented with NL:MPP8 and MPP8:NL were detected with the MPP8-specific antibody. **(D)** WT and Periphilin KO (PKO) control cells, along with PKO cells complemented with NL:Periphilin and Periphilin:NL were detected with the Periphilin-specific antibody. **(E)** Western blot of the Periphilin variants used in Fig. 1E were detected with the HA tag-specific mAb. Anti-β-actin used as a loading control in all panels.

Figure S2:

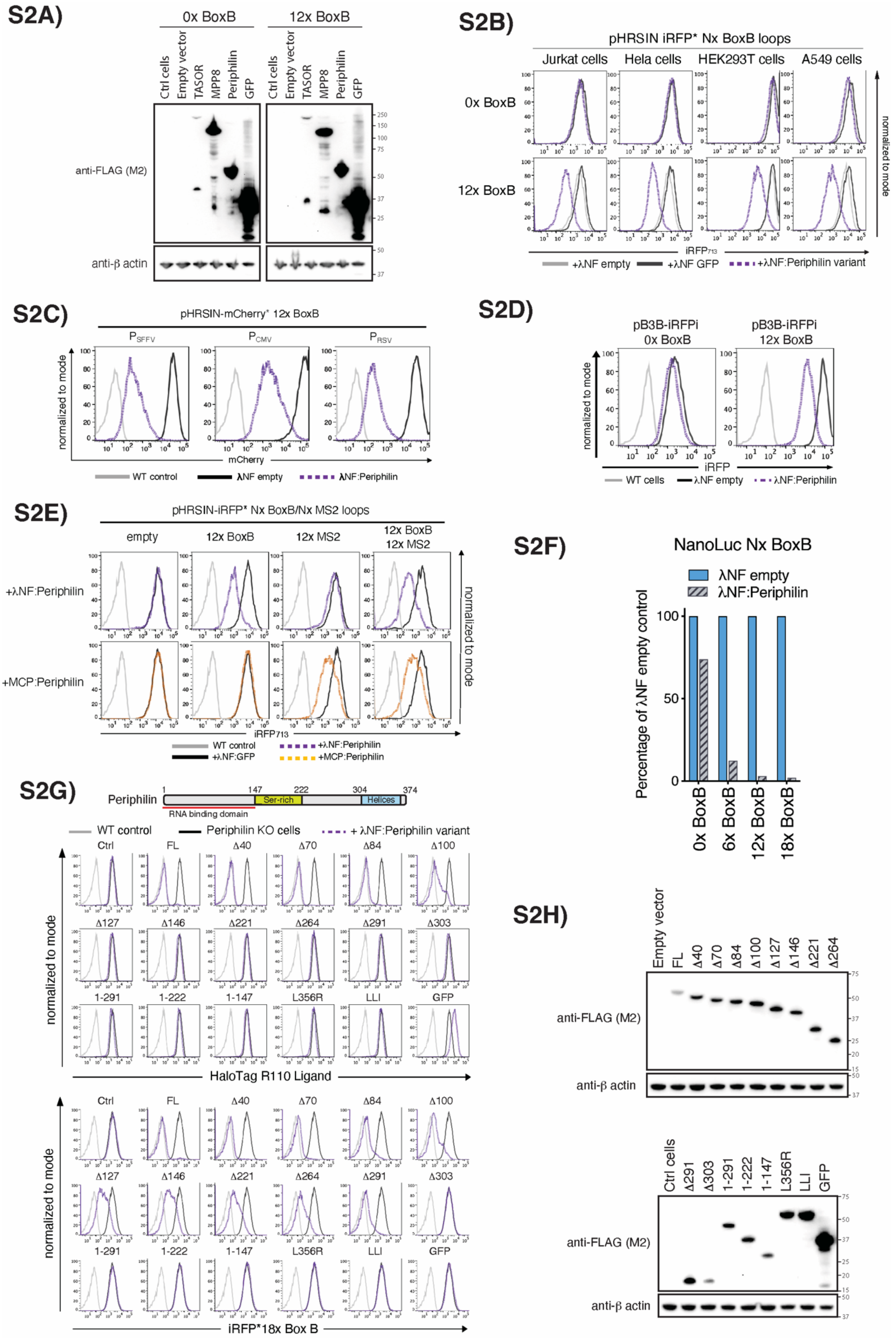
S2A)
0x BoxB
12x BoxB
Ctrl cells
Empty vector
TASOR
MPP8
Periphilin
GFP
anti-FLAG (M2)
anti-β actin
S2B)
pHRSIN iRFP* Nx BoxB loops
Jurkat cells
Hela cells
HEK293T cells
A549 cells
0x BoxB
12x BoxB
normalized to mode
iRFP713
+λNF empty
+λNF GFP
+λNF:Periphilin variant
S2C)
pHRSIN-mCherry* 12x BoxB
P_{SFFV}
P_{CMV}
P_{RSV}
normalized to mode
mCherry
WT control
λNF empty
λNF:Periphilin
S2D)
pB3B-iRFPi 0x BoxB
pB3B-iRFPi 12x BoxB
normalized to mode
iRFP
WT cells
λNF empty
λNF:Periphilin
S2E)
pHRSIN-iRFP* Nx BoxB/Nx MS2 loops
empty
12x BoxB
12x MS2
12x BoxB 12x MS2
+λNF:Periphilin
+MCP:Periphilin
normalized to mode
iRFP713
WT control
+λNF:GFP
+λNF:Periphilin
+MCP:Periphilin
S2F)
NanoLuc Nx BoxB
λNF empty
λNF:Periphilin
Percentage of λNF empty control
0x BoxB
6x BoxB
12x BoxB
18x BoxB
S2G)
Periphilin
Ser-rich
Helices
RNA binding domain
WT control
Periphilin KO cells
+ λNF:Periphilin variant
Ctrl
FL
Δ40
Δ70
Δ84
Δ100
Δ127
Δ146
Δ221
Δ264
Δ291
Δ303
1-291
1-222
1-147
L356R
LLI
GFP
normalized to mode
HaloTag R110 Ligand
iRFP*18x Box B
S2H)
Empty vector
anti-FLAG (M2)
anti-β actin
Ctrl cells

(A) Western blots of the samples in Fig. 2B were probed with a FLAG (M2)-specific mAb. Anti-β-actin was used as a loading control. **(B)** Flow cytometry of λNF empty and λNF:Periphilin transduced into either iRFP* 0x or 12x BoxB reporters in Jurkat, Hela, HEK293T and A549 cell lines, demonstrate that silencing by λN-BoxB tethering is independent of the cell line used. λNF empty (light grey), λNF:GFP (dark grey) or with λNF:Periphilin (dotted purple). **(C)** Flow cytometry of HeLa cells harbouring mCherry λN-BoxB tethering reporters driven by: SFFV, CMV or RSV promoters, demonstrates silencing is independent of the promoter used to drive the reporter. Wildtype control cells (light grey), λNF empty construct (dark grey) or with λNF:Periphilin (dotted purple). **(D)** Flow cytometry of λN-BoxB tethering in a PiggyBac transposon derived reporter system in HeLa cells, demonstrates that tethering-induced silencing by Periphilin is not dependent on lentiviral delivered reporter systems. Wildtype control cells (light grey), λNF empty construct (dark grey) or with λNF:Periphilin (dotted purple). **(E)** Flow cytometry of the λN-BoxB and MS2-MCP tethering systems in Jurkat Periphilin KO cells, demonstrates that tethering-induced silencing by Periphilin is effective with both tethering systems and not dependent on the type of RNA tethering system used. Wildtype control cells (light grey), λNF:GFP (dark grey), λNF:Periphilin (dotted purple) or MCP:Periphilin (dotted orange). **(F)** Luciferase quantification of a λN-NanoLuc BoxB tethering system in HeLa cells, showing increased λNF:Periphilin silencing with increasing BoxB stem loop number. **(G)** Complementation of Jurkat Periphilin KO cells, harbouring dual reporters: (i) NanoLuc:T2A:HaloTag reporter knocked into the ZNF37A locus (upper panel) and (ii) a lentiviral iRFP 18x BoxB tethering reporter (lower panel), with λNF(NLS):Periphilin deletion and mutant constructs. Mutants LLI and L356R disrupt dimerization and/or TASOR binding. Wildtype cells (light grey), reporter cells with λNF empty (dark grey) or with λNF:Periphilin deletions (dotted purple). **(H)** Western blot analysis of the samples in Fig. S2G detected with FLAG (M2)-specific mAb. Anti-β-actin was used as a loading control.

Figure S3:

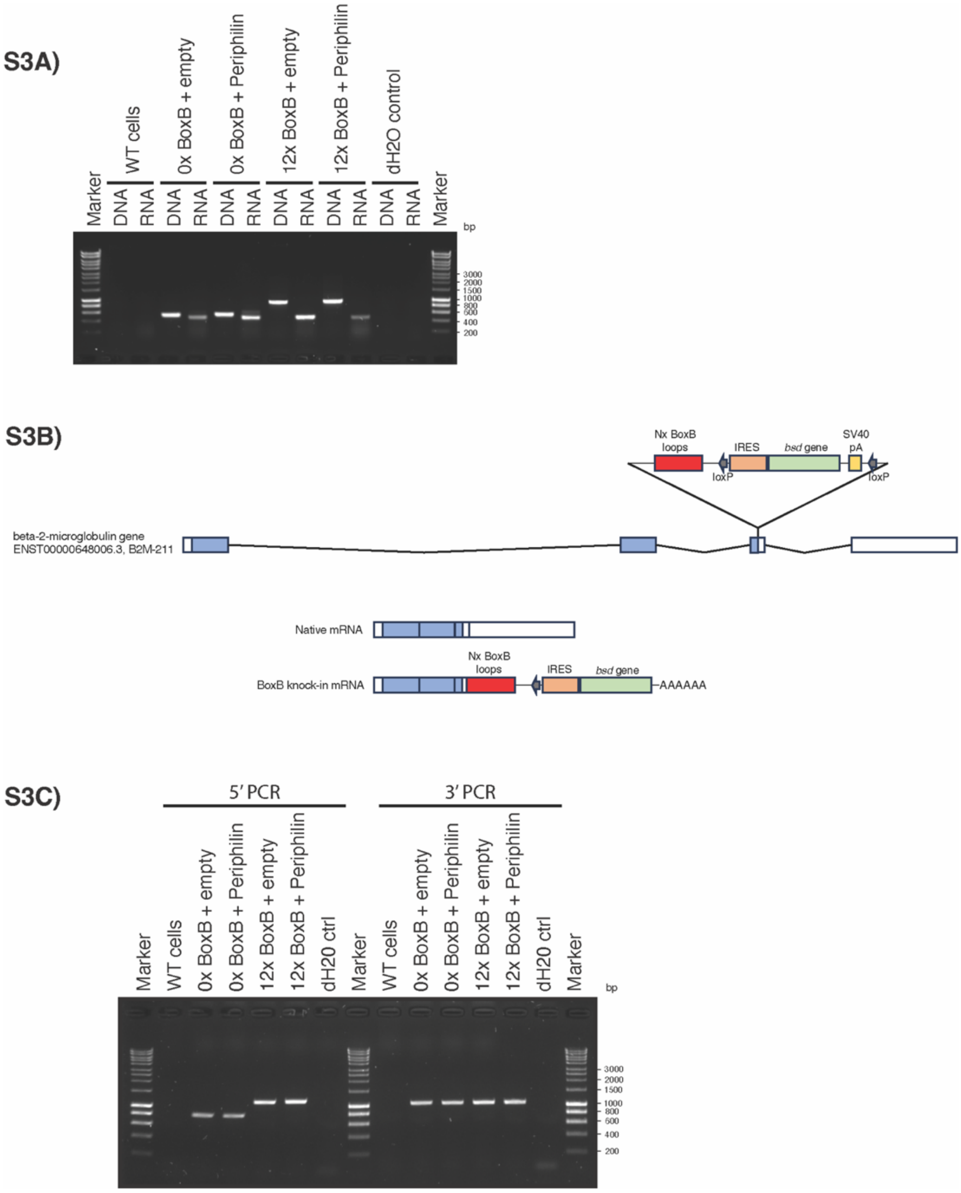

(A) TAE-agarose gel image of the samples in Fig. 3B and Fig. 3C confirms that λNF:Periphilin tethering does not disrupt splicing, using an intron-spanning PCR for gDNA and RT-PCR for mRNA. **(B)** Schematic of the beta-2-microglobulin (β2M) gene, showing the location within the 3'UTR, immediately downstream of the stop codon, for CRISPR/Cas9 knock-in of either an empty or 12x BoxB containing IRES-*bsd*-pA cassette. **(C)** TAE-agarose gel image confirming the presence of the IRES-*bsd*-pA cassette, using 5' and 3' flanking region PCRs, on a gDNA template from the samples in Fig. 3E and Fig. 3F.

Figure S4:

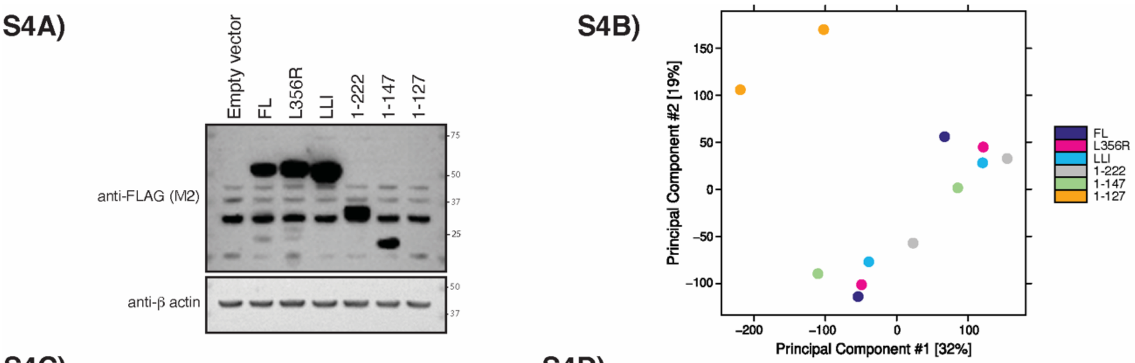

S4C)

Comparison	Up in FL	Down in FL	Total observed*
FL vs L356R	0	0	0
FL vs LLI	0	7	7
FL vs 1-222	1	7	8
FL vs 1-147	4	13	17
FL vs 1-127	1663	28	1691

* Total peaks compared = 31711

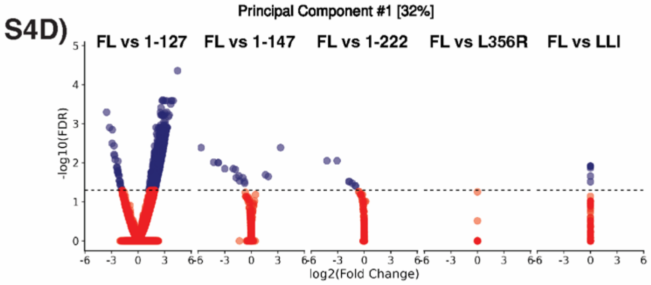

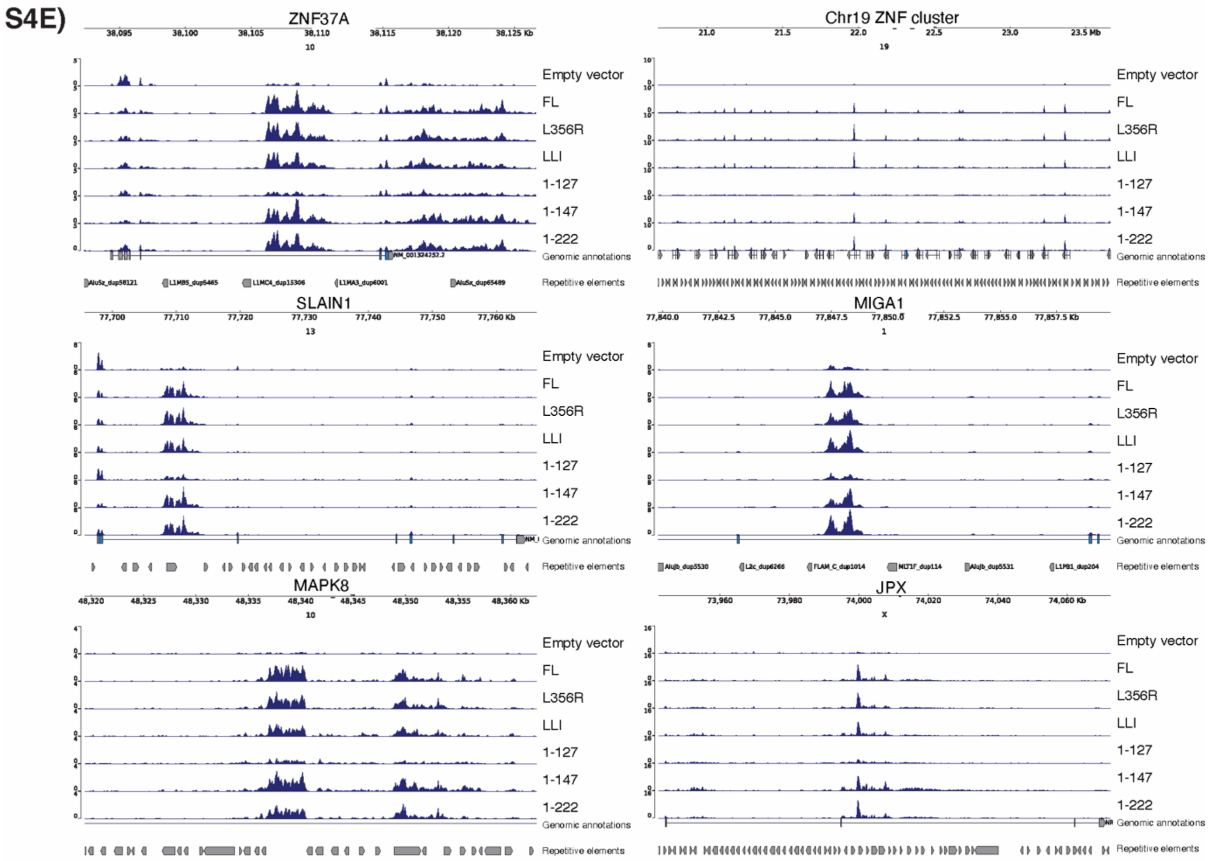

(A) Western blot analysis of the samples used in the Figure 4 Periphilin variant RIP-seq experiments probed with an HA-specific mAb. **(B)** PCA analysis of the Periphilin RIP-seq replicates. **(C)** The complete DiffBind table of the Periphilin RIP-seq samples, showing numbers of differential peaks relative to full length Periphilin (expansion of table, Fig. 4B).

(**D**) Volcano plots of the DiffBind determined differential peaks for each Periphilin variant compared to full length Periphilin. **(E)** Additional RIP-seq genome browser tracks showing further examples of Periphilin enrichment between the tested Periphilin variants. Values on y axis represent CPM.

**Description**	**Parent**	**Insert**	**Source**
**Lentiviral vectors:**			
pHRSIN-P_{SFFV}-GFP-WPRE-P_{GK}-Puro	-	-	Lehner Lab
pHRSIN-P_{SFFV}-GFP-WPRE-P_{GK}-Bsd	-	-	Lehner Lab
pHRSIN-P_{SFFV}-GFP-WPRE-P_{GK}-Hygro	-	-	Lehner Lab
pHRSIN-P_{SFFV}-(N-term)NanoLuc(BamHI-NotI)-WPRE-P_{GK}-Puro	pHRSIN-P_{SFFV}-GFP-WPRE-P_{GK}-Puro	Replaced GFP with an N-term (BamHI-NotI) NanoLuc tailed PCR product	Lehner Lab
pHRSIN-P_{SFFV}-(BamHI-NotI)NanoLuc(C-term)-WPRE-P_{GK}-Puro	pHRSIN-P_{SFFV}-GFP-WPRE-P_{GK}-Puro	Replaced GFP with an NanoLuc C-term (BamHI-NotI) tailed PCR product	Lehner Lab
pHRSIN-P_{SFFV}-NanoLuc^{STOP}(BamHI-NotI)-WPRE -P_{GK}-Hygro	pHRSIN-P_{SFFV}-GFP-WPRE-P_{GK}-Hygro	Replaced GFP with an NanoLuc- STOP (BamHI-NotI) tailed PCR product	Lehner Lab
pHRSIN-P_{SFFV}-(BamHI-NotI)HA:NLS:NanoLuc(C-term)-WPRE-P_{GK}-Puro	pHRSIN-P_{SFFV}-(BamHI-NotI)NanoLuc(C-term)-WPRE-P_{GK}-Puro	Replaced GFP with an phosphorylated and annealed HA:NLS oligos	Oligos
pHRSIN-P_{SFFV}-iRFP^{STOP}(BamHI-NotI)-WPRE-P_{GK}-Hygro	pHRSIN-P_{SFFV}-GFP-WPRE-P_{GK}-Hygro	Replaced GFP with an iRFP- STOP (BamHI-NotI) tailed PCR product	Lehner Lab
pHRSIN-P_{SFFV}-mCherry^{STOP}(BamHI-NotI)-WPRE-P_{GK}-Hygro	pHRSIN-P_{SFFV}-GFP-WPRE-P_{GK}-Hygro	Replaced GFP with an mCherry- STOP (BamHI-NotI) tailed PCR product	Lehner Lab
pHRSIN-P_{SFFV}-λN(NLS)FLAG(BamHI-NotI)-WPRE-P_{GK}-Bsd	pHRSIN-P_{SFFV}-GFP-WPRE-P_{GK}-Bsd	2 step cloning: (i) LambdaN (1-22) plus a GGGGSGGGS (BamHI-NotI) linker sequence, (ii) insertion of NLS:3xFLAG tag sequence into BamHI site	Oligos
pHRSIN-P_{SFFV}-MCP(BamHI-NotI)-WPRE-P_{GK}-Puro	pHRSIN-P_{SFFV}-GFP-WPRE-P_{GK}-Puro	Replaced GFP with an MCP(N55K)-linker-NLS(BamHI-NotI) PCR product	lentiMPH v2, EF1a-MS2-p65-HSF1-2A-Hygro-WPRE (Addgene #89308)
pHRSIN-P_{SFFV}-(BamHI-NotI)NLS:HA(C-term)-WPRE-P_{GK}-Bsd	pHRSIN-P_{SFFV}-GFP-WPRE-P_{GK}-Bsd	Replaced GFP with an phosphorylated and annealed (BamHI-NotI) NLS:HA oligos	Oligos
**LentiRev vectors: (The first expression cassette is in a reverse orientation)**			
LentiREV-(SV40pA-GFP-P_{SFFV})-P_{SV40}-Bsd-WPRE	-	-	Lehner Lab
LentiREV-(SV40pA-iRFPi(0xBoxB)-P_{SFFV})-P_{SV40}-Bsd-WPRE	LentiREV-(SV40pA-GFP-P_{SFFV})-P_{SV40}-Bsd-WPRE	Replaced GFP with an iRFPi (0x BoxB sequence)	This paper
LentiREV-(SV40pA-iRFPi(12xBoxB)-P_{SFFV})-P_{SV40}-Bsd-WPRE	LentiREV-(SV40pA-GFP-P_{SFFV})-P_{SV40}-Bsd-WPRE	Replaced GFP with an iRFPi (12x BoxB sequence)	This paper
LentiREV-(SV40pA-iRFP$_{713}$-P_{SFFV})-P_{SV40}-Bsd-WPRE	LentiREV-(SV40pA-GFP-P_{SFFV})-P_{SV40}-Bsd-WPRE	iRFP tailed PCR product	Lehner Lab
LentiREV-(SV40pA-0x BoxB-iRFP$_{713}$-P_{SFFV})-P_{SV40}-Bsd-WPRE (5'UTR intron)	LentiREV-(SV40pA-iRFP$_{713}$-P_{SFFV})-P_{SV40}-Bsd-WPRE	Insertion of a 0x BoxB containing intron into the 5' BamHI site	This paper
LentiREV-(SV40pA-12x BoxB-iRFP$_{713}$-P_{SFFV})-P_{SV40}-Bsd-WPRE (5'UTR intron)	LentiREV-(SV40pA-iRFP$_{713}$-P_{SFFV})-P_{SV40}-Bsd-WPRE	Insertion of a 12x BoxB containing intron into the 5' BamHI site	This paper
LentiREV-(SV40pA-iRFP$_{713}$-0x BoxB-P_{SFFV})-P_{SV40}-Bsd-WPRE (3'UTR intron)	LentiREV-(SV40pA-iRFP$_{713}$-P_{SFFV})-P_{SV40}-Bsd-WPRE	Insertion of a 0x BoxB containing intron into the 3' NotI site	This paper
LentiREV-(SV40pA-iRFP$_{713}$-12x BoxB-P_{SFFV})-P_{SV40}-Bsd-WPRE (3'UTR intron)	LentiREV-(SV40pA-iRFP$_{713}$-P_{SFFV})-P_{SV40}-Bsd-WPRE	Insertion of a 12x BoxB containing intron into the 3' NotI site	This paper
**PiggyBac vectors:**			
pB-CMVdox-ORF1-P2A-GFP-pUb-loxP-tetR-loxP-IRES-Blast	-	-	a gift from J. Wysocka
pB3B-MCS-SV40pA-P_{SV40}-Bsd-SV40pA	pB-CMVdox-ORF1-P2A-GFP-pUb-loxP-tetR-loxP-IRES-Blast	2 step cloning: (i) BsiWI-SalI fragmemnt replaced with an EcoRI-NotI-SalI MCS sequence, (ii) insertion of a P_{SV40}-EM7-Bsd-SV40pA PCR product into the EcoRV site	Oligos and pcDNA6/TR
pB3B-P_{SFFV}-iRFP^{STOP} 0x BoxB-SV40pA-P_{SV40}-EM7-Bsd-SV40pA	pB3B-MCS-SV40pA-P_{SV40}-Bsd-SV40pA	EcoRI (BsaI)-NotI (PspOMI) PCR fragments from LentiREV-(SV40pA-iRFPi(0xBoxB)-P_{SFFV})-PSV40-Bsd-WPRE into the EcRI-NotI sites	This paper
pB3B-P_{SFFV}-iRFP^{STOP} 12x BoxB-SV40pA-P_{SV40}-EM7-Bsd-SV40pA	pB3B-MCS-SV40pA-P_{SV40}-Bsd-SV40pA	EcoRI (BsaI)-NotI (PspOMI) PCR fragments from LentiREV-(SV40pA-iRFPi(12xBoxB)-P_{SFFV})-PSV40-Bsd-WPRE into the EcRI-NotI sites	This paper
pSFFViRFPf: tctagtatagt GGTCTC A aatt TGCAGCCCCGATAAAATAAAAG			
**Donor vectors:**			
pCR Blunt II-TOPO-5'ARM-0x BoxB(BamHI-NotI)-loxp-IRES-Bsd-SV40 pA-loxP-3'ARM	pCR Blunt II-TOPO	PCR joining of three overlapping fragments; (i) 350bp 5'ARM-(BamHI-NotI)-IRES gene block, (ii) pB3B Bsd-pA-loxP PCR fragment from pB3B, and (iii) loxP-350 bp 3'ARM PCR product.	Zero Blunt™ TOPO™ PCR Cloning Kit, Thermo Fisher
pCR Blunt II-TOPO-5'ARM-12x BoxB-loxp-IRES-Bsd-SV40 pA-loxP-3'ARM	pCR Blunt II-TOPO-5'ARM-0x BoxB(BamHI-NotI)-loxp-IRES-Bsd-SV40 pA-loxP-3'ARM	12x BoxB PCR fragment	This paper
**Lentiviral packaging and transposase vectors:**			
pCMVΔR8.9	-	-	Lehner Lab
pMD2.G	-	-	Lehner Lab
piggyBac-transposase expression plasmid; cat. no.PB210PA-1	-	-	System Biosciences
pSpCas9(BB)-2A-Puro (PX459)	-	-	Addgene, #48139

**Description**	**Name**	**Sequence**
nt NanoLuc (Bgl II-Not I)	ntNanoLucF:	ttgact agatct acc atggtcttcacactcgaagatttcgt
	ntNanoLucR:	tcataat gcggccgc ttcattcattca ggatcc gctgccgctgcc cgccagaatgcgttcgcaca
ct NanoLuc (Bam HI-PspOMI)	ctNanoLucF:	ttgact ggatcc tgaatgaatga gcggccgc a ggcagcggcagc gtcttcacactcgaagatttcgt
	ctNanoLucR:	ttgatg gggccc tca cgccagaatgcgttcgcaca
HA-NLS (NotI site of ct NL)	NotIHANLSf:	ggccgc T GGC AGC GGC TCC TACCCATACGATGTTCCAGATTACGCT GGA TCT GGC TCT CCCAAGAAGAAACGGAAAGT
	NotIHANLSr:	ggcC ACTTTCCGTTTCTTCTTGGG AGA GCC AGA TCC AGCGTAATCTGGAACATCGTATGGGTA GGA GCC GCT GCC Ggc
LambdaN-3xFLAG	lambdaNFor:	ttgact agatct acc ATGAATGCCCGGACTAGAAGGCGA
	lambdaNlinkerR:	tcataat gcggccgc ttcattcattca ggatcc CTGCAGTCTTCCAGATCCCCCA
	NLSFLAGf:	GATCa cccaagaagaagcggaaggtc GACTACAAAGACCATGACGGTGATTATAAA GATCATGACATCGATTACAAGGATGACGATGACAAG G
	NLSFLAGr:	GATCC CTTGTCATCGTCATCCTTGTAATCGATGTCATGATCTTTATAATCACCGTCATGGTCTTTGTAGTC gaccttccgcttcttcttggg t
iRFP STOP (BamHI-NotI)	iRFP_Ct_ncM2f:	atccgtaa GGTCTC a GATC ACC ATGgcggaaggctccgtcgcc
	iRFP_Ct_ncM2r:	agtatagt GCGGCCGC CTATTCAGTTA GGATCC tca tcactcttccatcacgc
NanoLuc STOP (BamHI-NotI)	NanoLuc_Ct_ncF: (BsaI)	atccgtaa GGTCTC a GATC ACC atggtcttcacactcgaagatttcg
	NanoLuc_Ct_ncR:	agtatagt GCGGCCGC CTATTCAGTTA GGATCC tca cgccagaatgcgttcgcac
iRFP intron (BsaI)	iRFPfor2:	atccgtaa GGTCTC a GATC acc ATG gcggaaggctccgtcgc
	iRFPi304r:	cagtaaagcggccgcatcaattactcaggatccagaaACTTACctgcgtcctttcgcatcgtg
	iRFPi305f:	gtaattgatgcggccgctttactgAcTCCCTTTTTTTTTTCAGgTttcatcggctcctggcatcg
	iRFPrev:	agtatagt GGTCTC a GGCC TCA ctcttccatcacgccga
5'UTR intron	5UTRiFor:	atccgtaa GGTCTC G gatc tgcgaaaggacgcaggtaagtttct
	5UTRiRev2:	atccgtaa GGTCTC G gatc caggagccgatgaaacctg
3'UTR intron	3UTRiFor2:	atccgtaa GGTCTC G ggcc tgcgaaaggacgcaggtaagtttct
	3UTRiRev2:	atccgtaa GGTCTC G ggcc ccaggagccgatgaaacctg
HUSH constructs	TASORfor:	acatgact ggatcc atggcgactgctgtggagacgg
	TASORdATGfor:	acatgact ggatcc gcgactgctgtggagacgg
	TASORrev2:	cacaagcgt gcggccgc tta TTTCTCTTGTGAATATGGCCTGGAAGAATC
	TASORdSTPrev2:	cacaagcgt gcggccgc TTTCTCTTGTGAATATGGCCTGGAAGAATC
	MPP8f:	atccgtaa GGATCC ACC atg gagcaggttgcggaggga
	MPP8dATGf:	atccgtaa GGATCC gagcaggttgcggagggagc
	MPP8r:	agtatagt GCGGCCGC tca ctgcagctgcactctgt
	MPP8dSTOPr:	agtatagt GCGGCCGC ctgcagctgcactctgtatg
	PPHLN1f:	atccgtaa GGATCC ACC atg gcttacagaagagacgaaatgtg
	PPHLN1dATGf:	atccgtaa GGATCC gcttacagaagagacgaaatgtggtc
	PPHLN1r:	agtatagt GCGGCCGC cta aaaaggctctccaaaatcttgagtgga
	PPHLN1dSTOPr:	agtatagt GCGGCCGC aaaaggctctccaaaatcttgagtgga
Periphilin mutants (BamHI-NotI)	PPHLN1f:	atccgtaa GGATCC ACC atg gcttacagaagagacgaaatgtg
	PPHLN1r:	agtatagt GCGGCCGC cta aaaaggctctccaaaatcttgagtgga
	PPHLN1dSTOPr:	agtatagt GCGGCCGC aaaaggctctccaaaatcttgagtgga
	PPHLN1_41f:	atccgtaa GGATCC ACC atg GTGCCAAAGAAACCACCACTGCT
	PPHLN1_71f:	atccgtaa GGATCC ACC atg GGCCGCAGTTTTTCTCATGATCGA
	PPHLN1_85f:	atccgtaa GGATCC ACC atg AGAGGAGATGAATCTGGTTATAGATGGA
	PPHLN_101f:	atccgtaa GGATCC ACC atg AGCAGGCAACCTGAATACAGGGA
	PPHLN1_128f:	atccgtaa GGATCC ACC atg TCTCCTTATAAAAGGGACAATAC TTTTTTCAGA
	PPHLN1_147f:	atccgtaa GGATCC ACC atg TCTCCACACAGCAGATCTGGTTCCA

	PPHLN1_222f:	atccgtaa GGATCC ACC atg TCAAAGGTGTTAGACAAACCCAGT
	PPHLN1_265f:	atccgtaa GGATCC ACC atg GGcTCCACAGCACCATTGTTTACT
	PPHLN1_292F:	atccgtaa GGATCC ACC atg AGTCAGCTAACCACTCGCTCTAAAGCA
	PPHLN1_304f:	atccgtaa GGATCC ACC atg AAAACCAAAGAGATTGAACAGGTTTAC
	PPHLN1_127r:	agtatagt GCGGCCGC CCGCTCTCTCGCATAATGGGA
	PPHLN1_127TGAr:	agtatagt GCGGCCGC tca CCGCTCTCTCGCATAATGGGA
	PPHLN1_147r:	agtatagt GCGGCCGC AGAATCCTTTCGGCCAACAGGT
	PPHLN1_147TGAr:	agtatagt GCGGCCGC tca AGAATCCTTTCGGCCAACAGGT
	PPHLN1_222r:	agtatagt GCGGCCGC TGATGAAGAAACTGCTGAACCACT
	PPHLN1_222TGAr:	agtatagt GCGGCCGC tca TGATGAAGAAACTGCTGAACCACT
	PPHLN1_291r:	agtatagt GCGGCCGC ATCTTCAAATAATTCTATCCCATGTGTTG
	PPHLN1_291TGAr:	agtatagt GCGGCCGC tca ATCTTCAAATAATTCTATCCCATGTGTTG
	PPHLN1_304r:	agtatagt GCGGCCGC TTTTGATGCTATTGCCTTGCTA
	PPHLN1_304TGAr:	agtatagt GCGGCCGC tca TTTTGATGCTATTGCCTTGCTAC
	PPHLN1_LLIf:	gatggtggtgaaaatgGCgattgaaaaagatccttcaGCagaaaagtctGCacagtttgcattgaggca
	PPHLN1 _LLIr:	gatctttttcaatcGCcattttcaccaccatc
	PPHLN1_L356Rr2:	ttcattcattca gcggccgc TCA aaaaggctctccaaaatcttgagtggaagtatcatactctgcaat gaaatgcttgCGttcttcaacaca
pB3B-MCS-SV40pA-PSV40-Bsd-SV40pA	pB3MCSf:	gtacg GAATTC ctaactagtttatgac GCGGCCGC G
	pB3MCSr:	TCGAC GCGGCCGC gtcataaactagttag GAATTC c
PCR from pCDNA6/tr	GibBsdF:	tggggggggggctgtccctgat ATCGGTGTGGAAAGTCCCCAGGCT
	GibBsdR:	tatattttcttgttatagat ATAAGATACATTGATGAGTTTGGACAAACCACA
	pB3fixF:	gtacAttaaagataatcatgtgtaaaattgacgcatgC
	pB3fixR:	GTACGcatgcgtcaattttacacatgattatctttaaT
Cloning iRFPi or Nli into pB3B	pSFFViRFPf: (BsaI)	tctagtatagt GGTCTC A aatt TGCAGCCCCGATAAAATAAAAG
	iRFPpspOMIr: (NotI)	tctagtatagt GGGCCC tcatcactcttccatcacgcc
B2M donor plasmid	B2m5ArmFor:	tactgtgggcagttgtaacacaatggt
	BsdpAFor:	GGTTTTCCTTTGAAAAACACGATAATACC atggccaagcctttgtctcaaga
	BsdpARev:	ATAACTTCGTATAATGTATGCTATACGAAGTTAT taagatacattgatgagtttggacaaaccaca
	B2M3ARMf:	ATAACTTCGTATAGCATACATTATACGAAGTTAT ttggattggatgaattccaaattctgct
	B2M3ARMr:	ccaaattctaagcagagtatgtaaattgga
N-term MCP PCR primers	MS2NLSfor:	tctctttgact AGATCT acc ATGgcttcaaactttactcagtt
	MS2NLSrev:	agtatagt GCGGCCGC CTATTCAGTTA GGATCC caccttcctctttttcttaggtccgct
iRFPi intron/splicing check	iRFPiCheckF:	acgatgagccgatccatatc
	iRFPiCheckR:	agatcatcacccgatcgaag
5' ARM b2m KI check	b2mOuterF:	agggaattgatttgggagag
	b2mIRESrev:	ggaactgcttccttcacgac
3' ARM b2m KI check	b2mOuterR:	cactgaggccctttacactg
	b2mBsdFor:	tcctgggatcaaagccatag
**Sequencing Primers:**		
iRFPi intron sequencing	iRFPiINSseqF:	cgcacaaccgtctgacgat
iRFPi intron sequencing	iRFPiINSseqR:	gctcgaggaagatgagctgat
iRFPi intron sequencing	iRFPiseqF:	accgtctgacgatcgccttg
NLi intron sequencing	NLiSeqF:	ggtaatcgacggggttacgc
Lentiviral vector sequencing	SFFV_F	GCAGTTTCTTAAGACCCATCAG
Lentiviral vector sequencing	WPRE_R	GGAGCAACATAGTTAAGAATACC

Description	Name	Sequence
**sgRNAs:**		
TASOR	sg1-TASOR	ACATACTTAGGTCCAAATGA
	sg2-TASOR	TTGCAGCCTTTATGAAGTTG
	sg3-TASOR	GTTTCCTTATAAAACAGTGC
MPP8	sg1-MPP8	GCAGGTTGCGGAGGGAGCAA
	sg2-MPP8	GGGGGTATGTGGAGGGGCCC
	sg4-MPP8	TTTGGCGTTTGGGCAGATAC
Periphilin	sg1-Periphilin	GAGACGATCATTCTGCAAGC
	sg2-Periphilin	CTTCCCATTATGCGAGAGAG
	sg3-Periphilin	AGACCGCTCTCTCGCATAAT
SETDB1	sg1-SETDB1	TGGATGCATCATCAAAGAGT
	sg2-SETDB1	AACTCTTTGATGATGCATCC
	sg3-SETDB1	GCTCTGAGGACGAATCTTCC
	sg4-SETDB1	CAGAACTCCAAAAGACCAGA
**shRNAs:**		
	shControl	GATCCGTTATAGGCTCGCAAAAGGTTCAAGAGACCTTTTGCGAGCCTATAACTTTTTTG
	shTASOR	GATCCGAGGAAGCTTGAGGATCTATTCAAGAGATAGATCCTCAAGCTTCCTCTTTTTTG
	shMPP8	GATCCAAGAAGACCCCGAGAAAGGTTCAAGAGACCTTTCTCGGGGTCTTCTTTTTTTTG
	shPeriphilin	GATCCAGCTAACCACTCGCTCTAATTCAAGAGATTAGAGCGAGTGGTTAGCTTTTTTG
**KI sgRNAs:**		
b2m BoxB knock-in	b2m sgRNAfor:	GAATTCATCCAATCCAAATG

Gene	Primer name	5' - 3' sequence
iRFP	iRFP1for:	cttcgatcgggtgatgatct
	iRFP1rev:	gcaggcctagttttgactcg
B2M intron 2	b2mIn2qPCR1f:	ccctgaatgagtcccatccc
	b2mIn2qPCR1r:	cagtatctcagcaggtgcca

**6x BoxB loops (internal B-N)**	Ramanathan et al. aagtccaagccctgaaaaagggcaagcttggccctgaaaaagggcaagcttggccctgaaaaagggcaagcttg. box B (nut R) sequence: gccctgaaaaagggc	
	Phosphorylated, annealed and ligated	
	boxBf1:	GATCaagccctgaaaaagggcaagcttggccctgaaaaagggcaagcttggccctgaaaaa gggcaagcttgggatcc
	boxBf2:	actgtcttttatagacgcggccgccaagccctgaaaaagggcaagcttggccctgaaaaagggc aagcttggccctgaaaaagggcaagctt
	boxBr2:	GGCCaagcttgccctttttcagggccaagcttgccctttttcagggccaagcttgccctttttcag ggcttggcggccgcgtctataaaa
	boxBr1:	gacagtggatcccaagcttgccctttttcagggccaagcttgccctttttcagggccaagcttg ccctttttcagggctt
**6x MS2 loops (internal B-N)**	PCR'd from the 2 oligos	
	6xMS2for:	tctctttgact AGATCT acatgaggatcacccatgtctgaaggttgactctagaaaacatgaggatcaccc atgtctgaacatcgactctagaaaacatgaggatcacccatgtcggatccactacaaaatagacgcggccgc
	6xMS2rev:	agtatagt GGGCCC gacatgggtgatcctcatgttttctagagtcgatgttcagacatgggtgatcctcat gttttctagagtcaaccttcagacatgggtgatcctcatgtggcggccgcgtctat
	MS2PCRfor:	ggtctctttgact AGATCT ac atgaggatca
	MS2PCRrev:	ggagtatagt GGGCCC gacatgggt
**B2M 12x BoxB donor sequence**	tactgtgggcagttgtaacacaatggtaagtatttgtgtatctaaacatagaagttgcagtaaaaatatgctattttaatcttatgagaccactgtcatatatacagtccatcattgaccaaaacatcatatcagcatttttttcttctaagattttgggagcaccaaagggatacactaacagg atatactctttataatgggtttggagaactgtctgcagctacttcttttaaaaaggtgatctacacagtagaaattagacaagtttggtaatgagatctgcaatccaaataaaataaattcattgctaaccttttttcttttcttttcaggtttgaagatgccgcatccggatcgcatggacgagct gtacaagtgaggatcaagccctgaaaaagggcaagcttggccctgaaaaagggcaagcttggccctgaaaaagggcaagcttgggatcaagccctgaaaaagggcaagcttggccctgaaaaagggcaagcttggccctgaaaaagggcaagcttgggatccactgtctttt atagacgcggccgccaagccctgaaaaagggcaagcttggccctgaaaaagggcaagcttggccctgaaaaagggcaagcttggccgccaagccctgaaaaagggcaagcttggccctgaaaaagggcaagcttggccctgaaaaagggcaagcttggccgcgactcta gagtcgacctgcagggccgccacataacttcgtatagcatacattatacgaagttatacccccccctaacgttactggccgaagccgcttggaataaggccggtgtgcgtttgtctatatgttattttccaccatattgccgtcttttggcaatgtgagggcccggaaacctggccctg tcttcttgacgagcattcctaggggtctttcccctctcgccaaaggaatgcaaggtctgttgaatgtcgtgaaggaagcagttcctctggaagcttcttgaagacaaacaacgtctgtagcgaccctttgcaggcagcggaaccccccacctggcgacaggtgcctctgcggccaa aagccacgtgtataagatacacctgcaaaggcggcacaaccccagtgccacgttgtgagttggatagttgtggaaagagtcaaatggctctcctcaagcgtattcaacaaggggctgaaggatgcccagaaggtaccccattgtatgggatctgatctggggcctcggtgcacat gctttacatgtgtttagtcgaggttaaaaaaacgtctaggccccccgaaccacggggacgtggttttcctttgaaaaacacgataataccatggccaagcctttgtctcaagaagaatccaccctcattgaaagagcaacggctacaatcaacagcatccccatctctgaagactaca gcgtcgccagcgcagctctctctagcgacggccgcatcttcactggtgtcaatgtatatcattttactgggggaccttgtgcagaactcgtggtgctgggcactgctgctgctgcggcagctggcaacctgacttgtatcgtcgcgatcggaaatgagaacaggggcatcttgagcc cctgcggacggtgccgacaggtgcttctcgatctgcatcctgggatcaaagccatagtgaaggacagtgatggacagccgacggcagttgggattcgtgaattgctgccctctggttatgtgtgggagggctaagcacttcgtggccgaggagcaggactgacacgtgctacga gatttcgattccaccgccgccttctatgaaaggttgggcttcggaatcgttttccgggacgccggctggatgatcctccagcgcggggatctcatgctggagttcttcgcccaccccaacttgtttattgcagcttataatggttacaaataaagcaatagcatcacaaatttcacaaata aagcatttttttcactgcattctagttgtggtttgtccaaactcatcaatgtatcttaataacttcgtatagcatacattatacgaagttatttggattggatgaattccaaattctgcttgcttgctttttaatattgatatgcttatacacttacactttatgcacaaaatgtagggttataataatgtt aacatggacatgatcttctttataattctactttgagtgctgtctccatgtttgatgtatctgagcaggttgctccacaggtagctctaggagggctggcaacttagaggtggggagcagagaattctcttatccaacatcaacatcttggtcagatttgaactcttcaatctcttgcactcaa agcttgttaagatagttaagcgtgcataagttaacttccaatttacatactctgcttagaatttgg	
**N-term MCP-linker-NLS**	atggcttcaaactttactcagttcgtgctcgtggacaatggtgggacaggggatgtgacagtggctccttctaatttcgctaatggggtggcagagtggatcagctccaactcacggagccaggcctacaaggtgacatgcagcgtcaggcagtctagtgcccagaagagaaagt ataccatcaaggtggaggtccccaaagtggctacccagacagtgggcggagtcgaactgcctgtcgccgcttggaggtcctacctgaacatggagctcactatcccaattttcgctaccaattctgactgtgaactcatcgtgaaggcaatgcaggggctcctcaaagacggtaat cctatcccttccgccatcgccgctaactcaggtatctacagcgctggaggaggtggaagcggaggaggaggaagcggaggaggaggtagcggacctaagaaaaagaggaaggtg	
